# Measuring commissioners’ willingness-to-pay for community based childhood obesity prevention programmes using a discrete choice experiment

**DOI:** 10.1186/s12889-020-09576-7

**Published:** 2020-10-12

**Authors:** Edward J. D. Webb, Elizabeth Stamp, Michelle Collinson, Amanda J. Farrin, June Stevens, Wendy Burton, Harry Rutter, Holly Schofield, Maria Bryant

**Affiliations:** 1 Leeds Institute of Health Sciences, University of Leeds, Leeds, UK; 2 Clinical Trials Research Unit, Leeds Institute of Clinical Trials Research, University of Leeds, Leeds, UK; 3 Department of Nutrition, Gillings School of Global Public Health, University of North Carolina, Chapel Hill, USA; 4 Department of Epidemiology, Gillings School of Global Public Health, University of North Carolina, Chapel Hill, USA; 5 Department of Social and Policy Sciences, University of Bath, Bath, UK; 6 Department of Health Sciences and the Hull York Medical School, University of York, Heslington, York, UK

**Keywords:** Childhood obesity, Parental education, Discrete choice experiment, Willingness-to-pay, Service commissioners

## Abstract

**Background:**

In the UK, rates of childhood obesity remain high. Community based programmes for child obesity prevention are available to be commissioned by local authorities. However, there is a lack of evidence regarding how programmes are commissioned and which attributes of programmes are valued most by commissioners. The aim of this study was to determine the factors that decision-makers prioritise when commissioning programmes that target childhood obesity prevention.

**Methods:**

An online discrete choice experiment (DCE) was used to survey commissioners and decision makers in the UK to assess their willingness-to-pay for childhood obesity programmes.

**Results:**

A total of 64 commissioners and other decision makers completed the DCE. The impact of programmes on behavioural outcomes was prioritised, with participants willing to pay an extra £16,600/year if average daily fruit and vegetable intake increased for each child by one additional portion. Participants also prioritised programmes that had greater number of parents fully completing them, and were willing to pay an extra £4810/year for every additional parent completing a programme. The number of parents enrolling in a programme (holding the number completing fixed) and hours of staff time required did not significantly influence choices.

**Conclusions:**

Emphasis on high programme completion rates and success increasing children’s fruit and vegetable intake has potential to increase commissioning of community based obesity prevention programmes.

## Background

The number of children who start school with obesity in the UK has risen to 9.7% [1]. There are inequalities in prevalence, with children living in the most deprived areas more likely to be overweight or obese [1, 2]. Childhood obesity often persists into adulthood [3, 4], and due to related health issues, children with obesity can experience consequences as older children or adults. The consequences include effects on both physical health, e.g. pre-diabetes or high blood pressure [5], and mental health [6]. The UK government has set a goal to halve childhood obesity by 2030 [7].

In the UK, local authorities (a level of regional government) have a mandate to improve the health of the community. As part of addressing this responsibility, commissioners within local authorities can choose to provide various public health programmes. Local strategies and health priorities influence the choice of programmes that are commissioned. Childhood obesity is prioritised in many areas, with prevention in the early years now recognised as a key strategy [8, 9]. Programmes targeting obesity prevention in preschool children vary in their approach, including interventions aiming to modify food preferences (e.g. through repeated exposure to fruit and vegetables [10, 11]), parent focused interventions (e.g. [12, 13]) and behaviour change interventions directly targeting children [14].

Local authorities have limited resources, and every pound spent on childhood obesity programmes is a pound not spent on other policy areas, so commissioners and others involved in the process must carefully assess whether programmes are cost-effective. However, effectiveness evidence for many obesity prevention programmes is lacking. Thus, judgements may be based on other factors, such as likely success of implementation as assessed by monitoring data [15].

Further, there is a lack of published evidence on the commissioning process and the factors prioritised by decision-makers [15, 16], without which it is difficult to assess whether scarce resources are used optimally in practice. The current study attempted to address part of that knowledge gap by measuring decision-makers’ willingness-to-pay (WTP) for individual components of childhood obesity programmes using a discrete choice experiment (DCE) [17–20]. Decision-makers could include commissioners themselves, people who make recommendations about commissioning and people who help implement services. Although some previous research has looked at the commissioning of existing childhood obesity programmes [16, 21], the multicomponent nature of such programmes makes it difficult to disentangle what aspects and outcomes are most prioritised.

This study does not make any judgements as to whether the elicited preferences of commissioners and other decision-makers are appropriate, or whether they are likely to represent a good use of scarce resources. Instead, this study’s aim is to measure decision-makers’ priorities and the amounts they are in theory willing to pay to achieve given outcomes.

This work was conducted as part of a wider piece of research examining parental engagement with Health Exercise and Nutrition for the Really Young (HENRY), an early years obesity prevention programme in the UK [22, 23].

## Methods

### Survey development

In a DCE, participants are presented with a series of choices between hypothetical options, in this case which programme to provide. The options are described in terms of a set of attributes (e.g. number of parents enrolled, annual running cost). The levels the attributes take are varied in each question, and statistical analysis of their responses reveals the trade-offs participants make, e.g. more effective programmes versus increased cost of provision. A strength of the DCE methodology is that it allowed preferences for many different programmes to be estimated, including hypothetical programmes which do not currently exist, but could plausibly do so in future [24].

The attributes and levels selected for the DCE were guided by items/outcomes reported in previous evaluations [15, 25]. Initial attribute and level selection was done through unstructured discussion between authors (EW and WB) with expertise in health economics and childhood obesity until consensus was reached. Attributes and levels were finalised using unstructured discussion between authors (EW, WB, MC, HS, MB) until reaching consensus. We included six attributes, each with three levels (Table 1).

**Table 1:** Attributes and levels

Attribute	Level 1	Level 2	Level 3
Average enrolment	6 parents	8 parents	10 parents
Average completion rate (attending at least 5 sessions)	50% (3/4/5^a^ parents)	75% (4.5/6/7.5^a^ parents)	80% (4.8/6.4/8^a^ parents)
Average additional portions of fruit & veg eaten per day	0.5 portions	1 portion	2 portions
Hours of staff time per week	4 h	8 h	12 h
Set-up cost	£15,000	£20,000	£30,000
Annual running cost	£15,000	£20,000	£30,000

Note. ^a^ depending on average enrolment

**Fig. 1:**
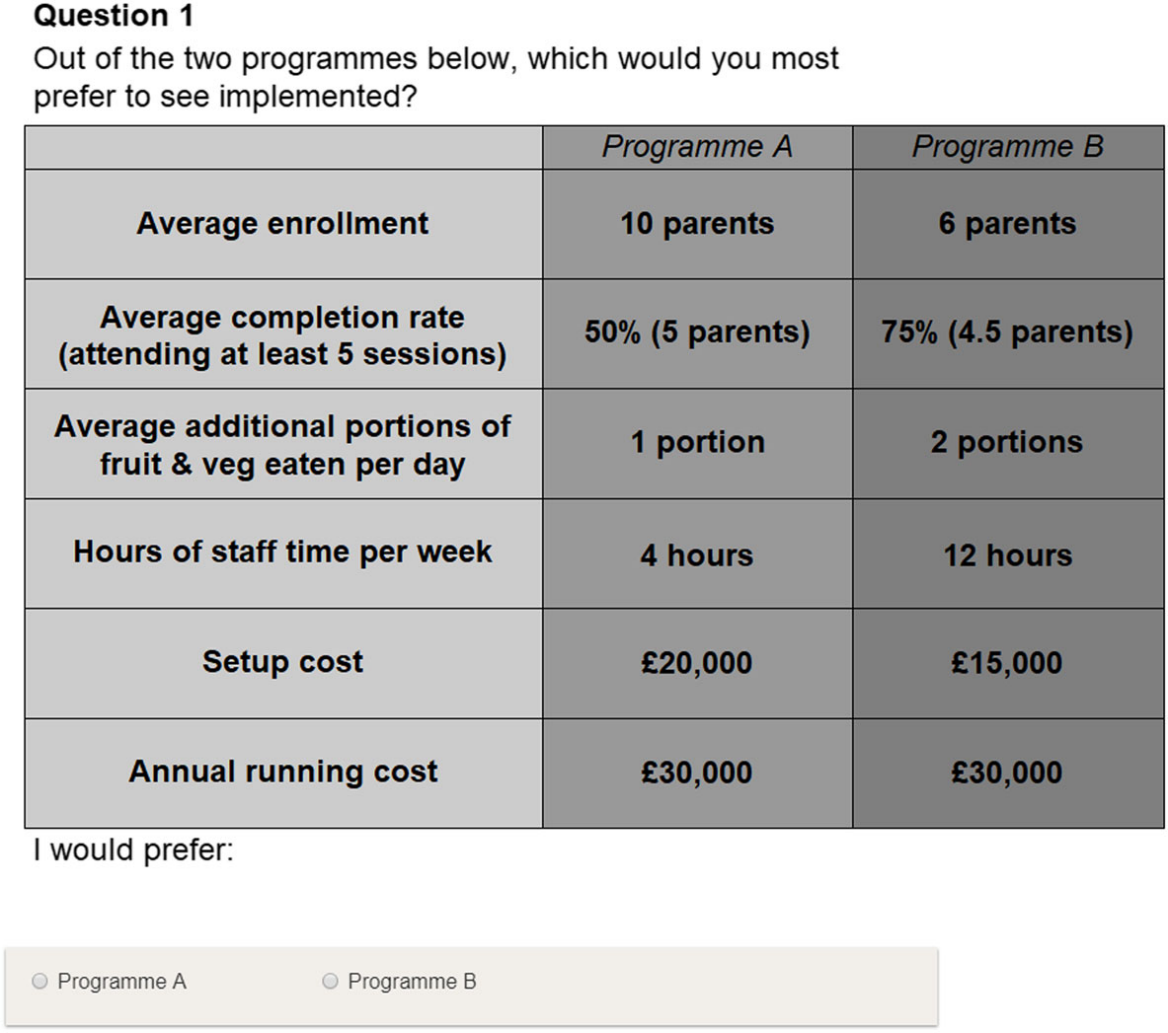
Example discrete choice experiment task

The survey was designed to offer participants a choice in each task between two programmes consisting of differing attributes, rather than providing three or more different programmes to minimise task difficulty. An example question is shown in Fig. 1. Within each programme, parents would attend one session per week for eight weeks. Around two to three cohorts of parents would be able to attend each year per centre. Survey participants were informed that parents were defined as having successfully completed a programme if they attended at least five out of eight sessions. Many DCEs include an opt-out option, e.g. selecting 11 to provide neither programme, and providing an opt-out may influence participants’ choices [26, 27]. An opt-out was not included in this survey, as it was felt that minimising respondent burden as much as possible was important given the target population.

The statistical design of the DCE (i.e. which attribute levels are presented to participants in each question) was generated using the Ngene software package.^1^ This program uses an algorithm to find a design that maximises D-efficiency, a standard statistic in the DCE literature, which may be thought of as a measure of how much information it is possible to gain from the responses [28]. More details, including the code used, are available in section 1 of the appendix. The final survey design, available in section 2 of the appendix, had 40 DCE tasks, which were split into four blocks so that each participant answered 10 questions, thus reducing the burden on respondents.

The survey was pilot tested with two commissioners who agreed that the attributes captured the most important features of childhood obesity programmes, that the survey was understandable, and that it presented an appropriate respondent burden.

### Survey recruitment and administration

The survey was promoted using multiple approaches, so it is not possible to know how many times a given person was contacted as they may have received information through multiple channels. Direct emails were sent to relevant organisations and contacts, with an initial email and one reminder sent. Relevant organisations and contacts were: 151 members of the Association of Directors of Children’s Services in the UK and seven organisations (Obesity Health Alliance, Association of Directors of Public Health, Children’s Commissioner, Local Government Association, Public Health England, Health and Social Care Committee, HENRY commissioners). The study was advertised on Twitter and at a national Obesity Congress in the UK (UKCO2019). Two organisations publicised the DCE in their newsletters (Obesity Health Alliance, Association of Directors of Public Health), two organisations tweeted the link to the DCE (Public Health England with over 200,000 followers, HENRY with over 2000 followers), and one organisation promoted it on LinkedIn (HENRY). The link was also tweeted from the account of the Clinical Trials Research Unit at the University of Leeds with over 400 followers, and re-tweeted by authors. Responses were collected between May 2019 and September 2019.

The survey was administered using Jisc Online Surveys. Following participant consent, survey instructions were provided. Participants then completed the DCE tasks, and finally they answered questions to help characterise their roles. Full details are provided in an example survey in the appendix. Ethical approval for the study was given by the Research Ethics Committee of the Faculty of Medicine and Health, University of Leeds. Respondents were not paid, but a £3 donation to a national children’s charity (National Society for the Prevention of Cruelty to Children, NSPCC) was made for every completed survey.

### Analysis

Responses were examined to see if participants “straight-lined” the DCE tasks, either by always selecting Programme A or by always selecting Programme B. Responses were analysed using a random utility theory framework. This standard approach [29] assumed participants chose the programme which gave them the highest utility. The utility of a programme was modelled as partly depending on the attributes of the programme, and partly random. The random component captured the influence of all factors in decision-making not explicitly included in the model.

Analysis of DCE responses only gives information about participants’ relative preferences, that is, they can only show how much they prefer an attribute relative to another attribute, not their absolute preference for that attribute. The model was estimated in WTP space [30, 31], a term which means that preferences for all other variables are measured relative to participants’ preferences for running costs. The result is that the model coefficients for all other variables can be directly interpreted as participants’ marginal WTP in running costs for other attributes. The magnitude of the coefficient on running costs has no direct interpretation and is termed the numeraire. However, a positive sign may be interpreted as a preference for programmes with a lower running cost. Further detail about model estimation is available in the appendix.

The attribute average completion rate was presented to participants using both the rate (e.g. 50% of parents completing) and the absolute number (e.g. 5 parents completing), as it was uncertain which would be more relevant for participants. However, as the completion rate and the number of parents completing cannot vary independently, it is not possible to include both in a single model. Thus two separate models were estimated, one including the completion rate, and one including the number of parents completing. The final preferred model was then chosen as the one which minimised the Bayesian information criterion (BIC), a measure of how well a model fits data [32].

The results of model estimation can be used to calculate participants’ WTP in annual running costs for programmes with any given enrolment, completion, portions of fruit and vegetables, hours of staff time and setup costs. WTP was calculated for a range of hypothetical programmes. WTP was also estimated for three real world programmes, with the numbers completing, portions of fruit and vegetables, etc. based on results reported in the literature [12, 13, 33]. Note that these are intended as illustrative examples for the purpose of giving context only; they are not presented as a rigorous evaluation of the programmes. The exact numbers used to calculate WTPs, and their sources, are provided in section 3 of the appendix.

Analysis was carried out using the Apollo choice modelling package [34] run on R version 3.3.1. Statistical significance was assessed at the 5% level after adjustment using Holm’s sequential Bonferroni correction [35].

## Results

A total of 112 people consented to participate, of whom 84 (75%) completed at least one DCE task, and 64 (57%) fully completed the survey. However, as responses were anonymous, it is possible that some individuals, who stopped part-way through the survey and then later returned to complete it, are counted twice. Only completed surveys were analysed. For completed surveys, answers to questions about respondent characteristics were examined for identical responses. None were found, which gives confidence that each completed survey is from a unique individual.

Respondents’ characteristics are summarised in Table 2. Over 80% of respondents were female. A large majority of respondents were of white ethnicity (84%), with the next largest ethnic group being Asian (9%). Respondents almost exclusively worked in England, with only 3% reporting working in Wales and none working in Scotland or Northern Ireland. Most areas in England were well represented, with at least five respondents from all areas except the East of England, which had four. The location with the greatest number of people working in it was London, with 13 participants.

**Table 2:** Participant demographics

	N (%)
Gender	
Male	11 (17.2)
Female	53 (82.8)
Other/prefer not to say	0 (0)
Ethnicity	
White	54 (84.4)
Mixed	1 (1.6)
Asian	6 (9.4)
Black	2 (3.1)
Arab	1 (1.6)
Other	0 (0)
Areas worked in^a^	
North West England	7 (10.9)
North East England	6 (9.4)
Yorkshire	7 (10.9)
West Midlands	7 (10.9)
East Midlands	5 (7.8)
East of England	4 (6.3)
South West England	6 (9.4)
South East England	8 (12.5)
London	13 (20.3)
Northern Ireland	0 (0)
Wales	1 (3.1)
Scotland	0 (0)
Non-UK	2 (1.6)
N	64

^a^Participants could select more than one response, so percentages do not sum to 100%

Respondents’ professional roles are summarised in Table 3. Over half reported helping to implement services, with 45% directly commissioning services, and a slightly lower percentage making recommendations about what to commission. The median length of time participants had been in their role was four years, with six participants (9%) having been in their position for under a year, and 12 participants (19%) having been in position for 10 years or more.

**Table 3:** Participants’ roles

Role^a^ (%)	Commission services	45.3
	Make recommendations to others about what services to commission	43.8
	Help implement services	56.2
	Other	6.2
Median length of time in role (years)		3.9
Minimum length of time in role (months)		1
Maximum length of time in role (years)		10+
N		64

^a^Participants could select more than one response, so percentages do not sum to 100%

No participant was observed to straight-line the DCE tasks. Of the two models estimated, the model with the number of parents completing had a lower BIC, at 568.8, than the model with completion rate, which had a BIC of 574.5. Models with lower BIC are judged to fit the data better, and to give a better explanation of participants’ choices. The model with number of parents completing was chosen as the final preferred model, and only results from this are presented here. Results from the alternative model are available on request to the corresponding author.

Estimation results are shown in Table 4. The coefficient on annual running costs was significantly positive, indicating that participants were significantly more likely to prioritise programmes with lower running costs. In addition, participants expressed a preference for parental completion of programmes over that of initial recruitment; participants were willing to pay an additional £4810 per year for each additional parent per cohort who completed attendance. In contrast, the coefficient on the average number enrolling had a magnitude around 25 times smaller, and was statistically insignificant. (Note that as these figures are for a single cohort, a single extra parent completing per cohort implies an extra two to three completing each year.)

**Table 4:** Estimation results

	Parameter Mean	Standard deviation
Average enrolment	0.173 (0.522)	0.324 (0.656)
Average number completing	4.81^a^ (0.813)	1.71 (0.793)
Average additional portions of fruit and veg eaten each day	16.6^a^ (2.29)	8.91 (1.83)
Hours of staff time per week	−0.588^a^ (0.253)	1.18 (0.264)
Setup cost (£1000 s)	−0.389^a^ (0.117)	0.516 (0.207)
Annual running cost (£1000 s)	0.149^a^ (0.0361)	0.0727 (0.0210)
N	64	

Note. Standard errors in parentheses

Parameters for attributes other than annual running cost represent marginal willingness-to-pay in £1000s, i.e. how much more participants are willing to pay for a programme with one extra unit of the attribute

^a^Indicates statistical significance at the 5% level after adjustment using Holm’s sequential Bonferroni correction [35]

Participants were significantly more likely to choose a programme that increased the number of portions of fruits and vegetables consumed by children. They were willing to pay an additional £16,600 if each child whose parent successfully completed a programme consumed on average one additional daily portion, holding all other variables constant. At 0.5 portions per completing parent, the lowest level presented to participants, they were willing to pay a total of £15,000 in setup costs and about £7500 in running costs for a programme with only two parents per cohort completing it.

Participants were also willing to pay around 3.5 times more for an improvement to a programme of one extra portion of fruit and vegetables eaten daily per child, holding all else constant, than for an improvement of one additional parent per cohort completing the programme, holding all else constant. More portions of fruit and vegetables per child mean more portions in total eaten if more parents complete the programme compared to fewer. Hence it was investigated whether participants were willing to pay an additional premium for programmes causing children to eat more portions of fruit and vegetables if more parents completed the programme. This was done by estimating a model with an interaction between responses for completion rate and fruits and vegetables. The interaction term was found to be insignificant (*p* = 0.697, full results available on request), hence there was no indication that participants were willing to pay any additional premium.

The coefficient on start-up costs was significant, negative, and has an absolute magnitude less than one. The interpretation is that participants prioritised running costs over start-up costs: participants were only willing to pay £0.39 in annual running costs for a £1 reduction in start-up cost. The additional number of staff hours required did not significantly influence choices, although the coefficient was negative, in line with expectations.

**Fig. 2:**
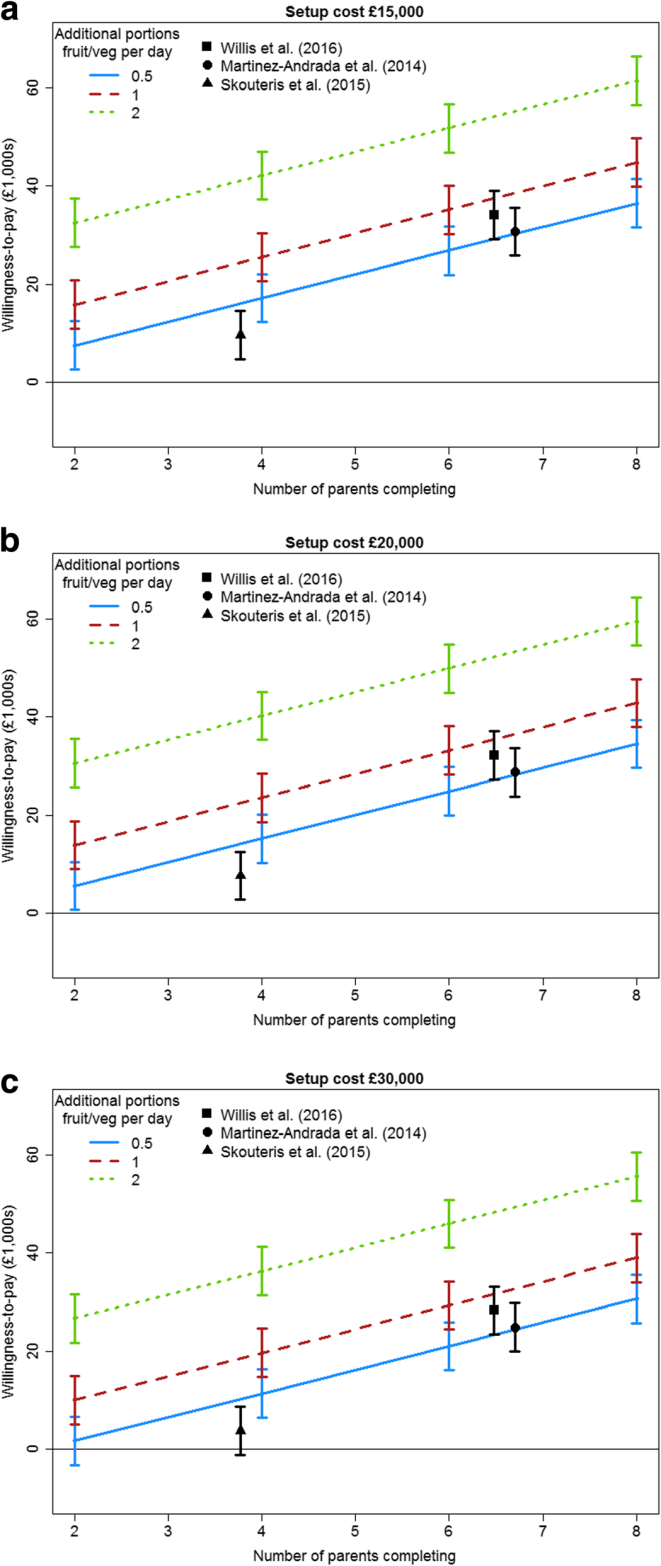
Estimated willingness to pay for different programmes. Note. Average completion rate = 66.7%, Staff hours = 8

Figure 2 illustrated WTP for a range of hypothetical programmes, based on the results in Table *4*. The attributes of average number of parents enrolling and hours of staff time were fixed at 66.7% and 8 h respectively, chosen as they are the midpoints of the attributes’ range. Also shown in the graphs are estimated WTP for three real world programmes.

## Discussion

Commissioners and related decision-makers in our study preferred programmes in which more parents completed them, with a WTP of over £4800 in running costs for each extra parent completing. In contrast, participants only expressed a weak preference for the number of parents enrolling. Thus the ability for programmes to retain participants is a key feature in whether decision-makers are willing to implement programmes. Recruiting more parents to a programme did not significantly alter our participants’ willingness to fund it unless it was possible to ensure that those parents also fully completed the programme.

Participants placed a high value on programmes that resulted in one extra portion of fruit and vegetables per day consumed by children, which may reflect the difficulties in achieving a full additional daily portion in a population. For example, several studies have shown that achieving a half extra portion per day is more achievable than one or two portions [36–39].

There are potential benefits of childhood obesity programmes not explicitly stated in the DCE attributes, for example physical activity or healthier eating habits. It is possible that some of the value placed on programmes by participants was due to these other benefits. Evidence for this is that there was considerable WTP for programmes with no effect on the number of portions of fruit and vegetables eaten. For example, with no additional portions and four parents completing, participants were still willing to pay £15,000 in setup costs and almost £9000 in running costs. Future research could usefully assess the value that decision-makers ascribe to outcomes such as encouraging exercise and improving eating habits.

Evidence from an ethnographic study within children’s centres [40] suggests that lack of staff time could be a barrier to implementing programmes from the perspective of service managers. This contrasts with the results of this study, that additional hours of staff time was not a major factor considered by commissioners and people supporting commissioning decisions over and above the broader financial costs, which were captured by other attributes. One potential reason for the difference between the perspectives of the participants from the two studies’ is that participants in the current study may not have been involved in the practical implementation of services. Future research could usefully examine differences in the perspectives of those involved in commissioning services and those managing them.

Setup costs were shown to substantially influence participants’ decision-making; however we found that annual running costs were valued more highly. This is expected, as the former is a one-off expense and the latter is a recurring expense, and so in the long run, the bulk of a programme’s cost will most likely be accounted for by annual running costs.

These results showed that participants were willing to pay large amounts of money for childhood obesity programmes. It is beyond the scope of this paper to assess whether this willingness to commit substantial funding is likely to result in an optimal use of resources, or whether the spending is cost-effective. However, it is hoped that the findings will inform a debate about whether current practice is in line with available evidence and what commissioning activity should focus on.

Previous studies have estimated the amount spent on behavioural weight management programmes for children, with figures of around £1300 [21] and £400 [21] per child completing. These figures are considerably lower than the WTP for the typical programme in our study, even when accounting for two to three cohorts attending per year. Yet the comparison is not straightforward, as the cited findings were for interventional programmes targeting overweight children, rather than the preventative programmes for parents evaluated in the current study. It may be that WTP was higher in this study due to prevention being prioritised over treatment, and future research could usefully explore this issue.

There is evidence that there is pressure on people involved in commissioning to reduce levels of childhood obesity [16]. While such pressure is not necessarily a bad thing, the high WTP found may reflect decision-makers feeling the need to take pro-active steps, even if they have limited impact. However, more research is required before being able to draw conclusions.

The outcomes of the hypothetical programmes were presented as certain to participants, so that when participants chose a programme they were guaranteed to achieve engagement and improvement in diet. For real-world programmes, achieving beneficial outcomes may be more uncertain, especially in the long term, in part due to a low evidence base [15, 16, 41, 42]. An alternative interpretation of a high WTP might be that the certainty associated with the hypothetical programmes meant they were perceived as representing a better use of resources than currently available programmes.

Although the behavioural outcomes of programmes were presented with certainty, the ultimate aim of the programmes was to reduce childhood obesity. No information on the impact of any behavioural change on obesity prevention was given to participants. Given the aforementioned low evidence base, the lack of information about the link between the amount of fruit and vegetables children eat and obesity may have made it difficult for participants to assess the value of programmes.

These results have highlighted some aspects of childhood obesity programmes that are prioritised by decision-makers and gives insights into what attributes they may wish to prioritise in monitoring and evaluation of programmes. However, this does not show which aspects are appropriate to emphasise in monitoring and evaluation in order to judge whether a programme represents a good use of resources. Further, our findings do not reflect the views of other stakeholders. Bryant et al. [15] conducted an exercise in which a range of stakeholders (including people involved in commissioning decisions) agreed on evaluation criteria for several public health interventions, including HENRY, a real-world programme similar to the hypothetical options presented to participants. The number of parents completing HENRY was not chosen as one of the top three evaluation criteria, in contrast to the preferences expressed by participants in this study. Further research could investigate potential differences in the priorities of commissioning decision-makers and other stakeholders.

We were able to apply our findings to estimate WTP for existing community-based obesity programmes, and found participants were willing to pay considerable amounts. For example, based on the findings of Willis et al. [36] participants would be willing to pay £15,000 in setup costs and £36,900 in annual running costs for the HENRY programme. Even with the MEND 2–4 programme, which had fewer than four parents completing, based on the results of Skouteris et al. [13] participants were willing to pay £15,000 in setup costs and £9600 in annual running costs. This exercise illustrates how the results could be used in future to assess whether new interventions are likely to be commissioned, analogous to the way in which DCEs have been included in predications of the uptake of medical treatments [43–45].

We are not aware of any previous DCEs that have targeted service commissioners and related decision-makers. Performing a DCE with this group raised potential challenges. For example, it was uncertain whether sufficient participants could be recruited, given a small target population with busy professional lives. It was also uncertain whether participants would find the choice tasks, involving deciding between providing alternative services, acceptable and meaningful. Participants’ preferences were in line with prior expectations (e.g. they preferred better outcomes and lower costs rather than the other way round), which is evidence that they interpreted the DCE tasks in the way they were intended and responded in a logical way. This study demonstrated that DCEs with service commissioners and related decision-makers are feasible, and future DCE studies could fruitfully elicit the preferences of similar populations.

This study has some limitations. It was not possible to assess how representative the sample was of all UK decision-makers, as almost all participants worked in England (albeit with representation from all areas of England). The sample was also self-selected and not randomly sampled, which means that participants may not be representative of the target population. For example, individuals who place a greater emphasis on childhood obesity prevention might both be more likely to value programmes and more likely to complete the survey. Over half of respondents did not directly commission services, meaning that although the results were indicative of the preferences of those supporting commissioning decisions in practice, they did not necessarily make funding decisions for implementation of services.

Our study sample size was small compared to many other similar studies [17]. The total of 64 participants is also somewhat lower than rules-of-thumb in the DCE literature, for example Johnson and Orme [46] recommend 75 and Lancsar and Louviere [47] recommend 80 given the survey design used here. However, the sample is likely to represent a reasonable fraction of all possible respondents in the UK given the specialised nature of the target population. In particular, it is likely to represent a large fraction of commissioners given over half of participants reported performing that role. Additionally, the numbers recruited allowed robust, advanced statistical models to be estimated.

It is often considered good practice to work closely with stakeholders representing the participant population when developing DCE surveys and deciding attributes [48, 49]. Our input was limited to consultation with two commissioners and obesity experts during the survey development. This may be justified by the fact that our attributes had largely been chosen from previous literature in this area [15, 23, 36]; though it may be a limitation that the construction of attributes and levels was heavily influenced by existing literature on a specific programme. In addition, participants were professionals, rather than patients, making it easier for authors to assess how appropriate the language of attributes and levels were for the target population.

This study presents participants’ stated preferences (i.e. statements about what they would do in a hypothetical decision-making situation). Concerns have been raised over how externally valid WTP estimates based on stated preference are (e.g. [50, 51]). For example, some studies have found that WTP estimates are subject to hypothetical bias [52, 53], meaning that individuals overstate how much they would pay compared to a real situation. This could be a reason that the WTPs measured here exceed previous estimates of the amounts spent on programmes in reality [16, 21]. Other studies suggest that the range of levels in a DCE can affect WTP [54], for example presenting a higher range of costs may increase WTP.

## Conclusions

This study has demonstrated that people involved in commissioning services state that they are willing to pay substantial amounts for the provision of programmes to prevent childhood obesity in preschool settings. In particular, they may be willing to pay more for programmes that have higher numbers of parents fully completing and successfully result in children eating more portions of fruit and vegetables.

Future research could build on the current findings. It would be beneficial to combine the stated preference data gathered here with real-world data, as Buckell and Hess [48] and Wuepper, Clemm and Wree [49] have done, in order to improve the external validity of findings. It would also be interesting to compare the preferences of commissioners to the preferences of those implementing services on the ground, as well as service users.

## Supplementary information

**Additional file 1.** Survey statistical design, model estimation, calculating willingness to pay for real world programmes.

**Additional file 2.** Survey on childhood obesity programmes. Example survey.

## Data Availability

Data is not publically available, as consent for this was not obtained. However, data may be made available on a case by case basis via a formal data sharing agreement either by contacting Leeds Institute of Health Sciences, University of Leeds, UK, or by contacting the corresponding author directly.

## Abbreviations

DCE: Discrete choice experiment
WTP: Willingness-to-pay
HENRY: Health Exercise and Nutrition for the Really Young
MEND: Mind, Exercise, Nutrition … Do It!
BIC: Bayesian information criterion

## Footnotes

1 ©ChoiceMetrics
