# Supplementary material for: Measuring commissioners’ willingness-to-pay for community based childhood obesity prevention programmes using a discrete choice experiment
Source: BMC Public Health. 2020 Oct 12;20:1535. doi: 10.1186/s12889-020-09576-7 (PMC7549208; doi:10.1186/s12889-020-09576-7)
Supplement: Supplementary file 1 — Additional file 1. Survey statistical design, model estimation, calculating willingness to pay for real world programmes. [file 12889_2020_9576_MOESM1_ESM.docx]

**Appendix**

1. Survey statistical design

Small positive (0.001) priors were assumed for enrolment, completion rate and fruit and vegetables, and small negative (-0.001) priors were assumed for staff time, setup cost and running cost. Dominated alternatives were excluded.

NGene code:

Design

;alts = alt1, alt2

;rows = 40

;block = 4

;eff = (mnl,d)

;rep = 500

;rdraws = halton(250)

;alg = mfederov

;reject:

alt1.enrollment>=alt2.enrollment and alt1.completion>=alt2.completion and alt1.fruitnveg>=alt2.fruitnveg and alt1.stafftime<=alt2.stafftime and alt1.setupcost<=alt2.setupcost and alt1.runningcost<=alt2.runningcost,

alt2.enrollment>=alt1.enrollment and alt2.completion>=alt1.completion and alt2.fruitnveg>=alt1.fruitnveg and alt2.stafftime<=alt1.stafftime and alt2.setupcost<=alt1.setupcost and alt2.runningcost<=alt1.runningcost

;model:

U(alt1) = b_enrollment*enrollment[6,8,10] + b_completion*completion[50,70,80] + b_enrollment_x_completion*enrollment*completion + b_fruitnveg*fruitnveg[0.5,1,2] + b_stafftime*stafftime[4,8,12] + b_setupcost*setupcost[15,20,30] + b_runningcost*runningcost[15,20,30]/

U(alt2) = b_enrollment*enrollment + b_completion*completion + b_enrollment_x_completion*enrollment*completion + b_fruitnveg*fruitnveg + b_stafftime*stafftime + b_setupcost*setupcost + b_runningcost*runningcost

$

Design

| Version | Task | Alternative | Enrolment | Completion rate | Fruit and vegetables | Staff time (hours) | Setup cost (£1000s) | Running cost (£100s) |
| --- | --- | --- | --- | --- | --- | --- | --- | --- |
| 1 | 1 | 1 | 6 | 0.8 | 2 | 12 | 15 | 30 |
| 1 | 1 | 2 | 10 | 0.5 | 0.5 | 8 | 30 | 15 |
| 1 | 2 | 1 | 6 | 0.5 | 0.5 | 8 | 15 | 30 |
| 1 | 2 | 2 | 10 | 0.5 | 2 | 8 | 30 | 15 |
| 1 | 3 | 1 | 10 | 0.8 | 0.5 | 4 | 15 | 20 |
| 1 | 3 | 2 | 6 | 0.8 | 2 | 12 | 30 | 20 |
| 1 | 4 | 1 | 6 | 0.8 | 0.5 | 4 | 15 | 15 |
| 1 | 4 | 2 | 10 | 0.5 | 2 | 12 | 30 | 30 |
| 1 | 5 | 1 | 6 | 0.5 | 2 | 12 | 30 | 30 |
| 1 | 5 | 2 | 10 | 0.8 | 1 | 4 | 15 | 15 |
| 1 | 6 | 1 | 6 | 0.5 | 2 | 12 | 15 | 15 |
| 1 | 6 | 2 | 6 | 0.8 | 0.5 | 12 | 30 | 30 |
| 1 | 7 | 1 | 10 | 0.5 | 0.5 | 12 | 30 | 30 |
| 1 | 7 | 2 | 6 | 0.8 | 0.5 | 4 | 20 | 15 |
| 1 | 8 | 1 | 10 | 0.5 | 1 | 4 | 30 | 15 |
| 1 | 8 | 2 | 10 | 0.8 | 2 | 8 | 15 | 30 |
| 1 | 9 | 1 | 6 | 0.5 | 2 | 4 | 15 | 15 |
| 1 | 9 | 2 | 6 | 0.8 | 0.5 | 4 | 30 | 30 |
| 1 | 10 | 1 | 6 | 0.5 | 2 | 12 | 30 | 15 |
| 1 | 10 | 2 | 10 | 0.5 | 0.5 | 12 | 15 | 20 |
| 2 | 1 | 1 | 10 | 0.8 | 1 | 4 | 30 | 30 |
| 2 | 1 | 2 | 10 | 0.5 | 1 | 12 | 15 | 20 |
| 2 | 2 | 1 | 6 | 0.5 | 2 | 4 | 20 | 30 |
| 2 | 2 | 2 | 6 | 0.8 | 0.5 | 12 | 30 | 15 |
| 2 | 3 | 1 | 6 | 0.8 | 1 | 12 | 15 | 30 |
| 2 | 3 | 2 | 10 | 0.8 | 2 | 4 | 30 | 15 |
| 2 | 4 | 1 | 6 | 0.7 | 2 | 4 | 15 | 30 |
| 2 | 4 | 2 | 6 | 0.5 | 0.5 | 12 | 30 | 15 |
| 2 | 5 | 1 | 10 | 0.8 | 2 | 12 | 30 | 15 |
| 2 | 5 | 2 | 6 | 0.5 | 1 | 4 | 15 | 30 |
| 2 | 6 | 1 | 10 | 0.8 | 0.5 | 4 | 20 | 15 |
| 2 | 6 | 2 | 10 | 0.5 | 2 | 12 | 20 | 30 |
| 2 | 7 | 1 | 10 | 0.7 | 0.5 | 4 | 15 | 30 |
| 2 | 7 | 2 | 6 | 0.7 | 2 | 4 | 30 | 15 |
| 2 | 8 | 1 | 6 | 0.8 | 2 | 8 | 15 | 30 |
| 2 | 8 | 2 | 6 | 0.5 | 0.5 | 8 | 30 | 15 |
| 2 | 9 | 1 | 10 | 0.5 | 1 | 12 | 30 | 30 |
| 2 | 9 | 2 | 10 | 0.8 | 0.5 | 4 | 20 | 15 |
| 2 | 10 | 1 | 6 | 0.5 | 1 | 4 | 15 | 30 |
| 2 | 10 | 2 | 6 | 0.8 | 2 | 12 | 20 | 15 |
| 3 | 1 | 1 | 6 | 0.5 | 1 | 12 | 15 | 30 |
| 3 | 1 | 2 | 10 | 0.8 | 2 | 4 | 20 | 15 |
| 3 | 2 | 1 | 6 | 0.8 | 0.5 | 12 | 30 | 30 |
| 3 | 2 | 2 | 8 | 0.5 | 2 | 4 | 15 | 15 |
| 3 | 3 | 1 | 10 | 0.5 | 1 | 12 | 15 | 15 |
| 3 | 3 | 2 | 10 | 0.8 | 2 | 8 | 30 | 30 |
| 3 | 4 | 1 | 10 | 0.8 | 2 | 12 | 15 | 15 |
| 3 | 4 | 2 | 6 | 0.8 | 1 | 4 | 30 | 15 |
| 3 | 5 | 1 | 10 | 0.5 | 2 | 4 | 20 | 15 |
| 3 | 5 | 2 | 6 | 0.8 | 0.5 | 12 | 15 | 30 |
| 3 | 6 | 1 | 6 | 0.5 | 2 | 12 | 20 | 15 |
| 3 | 6 | 2 | 8 | 0.7 | 0.5 | 4 | 30 | 30 |
| 3 | 7 | 1 | 8 | 0.8 | 1 | 12 | 30 | 15 |
| 3 | 7 | 2 | 10 | 0.5 | 0.5 | 4 | 15 | 30 |
| 3 | 8 | 1 | 6 | 0.7 | 2 | 4 | 15 | 15 |
| 3 | 8 | 2 | 10 | 0.8 | 0.5 | 12 | 30 | 15 |
| 3 | 9 | 1 | 6 | 0.5 | 2 | 4 | 30 | 30 |
| 3 | 9 | 2 | 8 | 0.8 | 1 | 12 | 15 | 15 |
| 3 | 10 | 1 | 10 | 0.5 | 2 | 4 | 30 | 30 |
| 3 | 10 | 2 | 6 | 0.5 | 0.5 | 8 | 20 | 15 |
| 4 | 1 | 1 | 6 | 0.8 | 2 | 4 | 15 | 30 |
| 4 | 1 | 2 | 8 | 0.7 | 0.5 | 12 | 30 | 15 |
| 4 | 2 | 1 | 10 | 0.5 | 0.5 | 4 | 15 | 20 |
| 4 | 2 | 2 | 10 | 0.8 | 2 | 12 | 30 | 30 |
| 4 | 3 | 1 | 6 | 0.8 | 0.5 | 4 | 30 | 20 |
| 4 | 3 | 2 | 10 | 0.8 | 2 | 12 | 20 | 30 |
| 4 | 4 | 1 | 6 | 0.5 | 1 | 4 | 30 | 30 |
| 4 | 4 | 2 | 8 | 0.8 | 2 | 12 | 15 | 15 |
| 4 | 5 | 1 | 10 | 0.5 | 0.5 | 12 | 15 | 30 |
| 4 | 5 | 2 | 6 | 0.5 | 1 | 4 | 30 | 20 |
| 4 | 6 | 1 | 6 | 0.8 | 2 | 12 | 15 | 15 |
| 4 | 6 | 2 | 10 | 0.8 | 0.5 | 12 | 20 | 30 |
| 4 | 7 | 1 | 6 | 0.5 | 0.5 | 12 | 20 | 20 |
| 4 | 7 | 2 | 10 | 0.5 | 2 | 4 | 20 | 30 |
| 4 | 8 | 1 | 10 | 0.7 | 2 | 4 | 30 | 30 |
| 4 | 8 | 2 | 6 | 0.8 | 0.5 | 12 | 15 | 15 |
| 4 | 9 | 1 | 6 | 0.5 | 1 | 12 | 20 | 30 |
| 4 | 9 | 2 | 10 | 0.5 | 0.5 | 4 | 30 | 15 |
| 4 | 10 | 1 | 10 | 0.8 | 2 | 12 | 15 | 20 |
| 4 | 10 | 2 | 6 | 0.5 | 1 | 4 | 30 | 15 |

1. Model estimation

The utility participant $i$ gave to programme $j$ was assumed to take the form

$$u_{ij}=-\beta_{i}^{rc}x_{j}^{rc}+\beta_{i}^{rc}\boldsymbol{\beta}_{\boldsymbol{i}}\boldsymbol{x}_{\boldsymbol{j}}+\varepsilon_{ij}$$

where $\beta_{i}^{rc}$ gives $i$’s preference for running cost, $x_{j}^{rc}$ is the running cost of programme $j$, $\boldsymbol{\beta}_{\boldsymbol{i}}$ is a vector giving $i$’s preference for all other attributes, $\boldsymbol{x}_{\boldsymbol{j}}$ is a vector giving the levels of all other attributes of $j$, and $\varepsilon_{ij}$ is a random error term following an extreme value distribution.

Mixed logit models were estimated using simulated maximum likelihood with 1,000 Halton draws. All parameters were modelled as normally distributed. Models were estimated using the Apollo choice modelling package for R [34].

1. Calculating willingness to pay for real world programmes

Calculations for all programmes assumed 8 staff hours.

*Willis et al. [36]*

Willis et al. report a non-randomised service evaluation of the HENRY programme in the UK.

| Attribute | Number | Source |
| --- | --- | --- |
| Average enrolment | 9 | *“…[HENRY] is delivered by trained facilitator pairs to groups of 8-10 parents”* (p.102). Midpoint of 9 taken. |
| Average number completing | 6.48 | 72% completion rate (p.104) with 9 enrolling means 6.48 completing. |
| Average additional portions of fruit & veg eaten per day | 0.8 | Table 3: 3.4 portions eaten at start of programme, 4.2 eaten at end. |

*Martínez-Andrade et al. [12]*

Martínez-Andrade et al. report the results of a trial of Creciendo Sanos, a pilot intervention in Mexico. Participants attended six weekly two hour sessions giving education on nutrition and physical activity.

| Attribute | Number | Source |
| --- | --- | --- |
| Average enrolment | 10 | *“Each group was formed with approximately 10 parent-child pairs”* (p.5). |
| Average number completing | 6.7 | 67% attended at least 5 workshops (p.9), 10 enrolling means 6.7 completing. |
| Average additional portions of fruit & veg eaten per day | 0.53 | Table 4: For parents attending at least 5 sessions, 6.6 extra servings of vegetables per week, 2.9 fewer servings of fruit per week, (6.6-2.9)/7=0.53 |

*Skouteris et al. [13]*

Skouteris et al. report the results of a randomised controlled trial of the MEND (Mind, Exercise, Nutrition . . . Do It!) 2–4 programme in Australia. Participants attended 10 weekly 90 minute workshops on nutrition, physical activity, parenting and lifestyle behaviours.

| Attribute | Number | Source |
| --- | --- | --- |
| Average enrolment | 4.6 | *“An average of 4.6 families participated in each programme.”* (p.6). |
| Average number completing | 3.77 | 82% attended at least 7 sessions (p.6), 4.6 enrolling means 3.77 completing. |
| Average additional portions of fruit & veg eaten per day | 0.11 | Table S1: Post-intervention 0.35 more portions of veg, 0.24 fewer portions of fruit. |
