# Supplementary material for: Measuring commissioners’ willingness-to-pay for community based childhood obesity prevention programmes using a discrete choice experiment
Source: BMC Public Health. 2020 Oct 12;20:1535. doi: 10.1186/s12889-020-09576-7 (PMC7549208; doi:10.1186/s12889-020-09576-7)
Supplement: Supplementary file 2 — Additional file 2. Survey on childhood obesity programmes. Example survey. [file 12889_2020_9576_MOESM2_ESM.pdf]

UNIVERSITY OF LEEDS

Survey on childhood obesity programmes

Consent

To begin the survey, please indicate that you consent to taking part.

- [ ] I confirm I have read the above information about how my data will be handled.
- [ ] I understand that any information I provide will be anonymous and stored securely.
- [ ] I give permission for my responses to be shared with other researchers provided the appropriate data sharing arrangements are in place, and for my responses to be re-analysed and used in relevant future research projects.
- [ ] I agree to take part in the survey.

Instructions

In the first part of the survey, we are going to show you examples of hypothetical programmes targeted at parents of small children. Each programme provides parents with practical guidance and help in improving parenting skills surrounding healthy eating and exercise. This guidance is delivered in group sessions, with one session per week over 8 weeks.

We are going to give you a series of 10 choices between two hypothetical programmes. Each is described in terms of 6 features:-

Programme feature	Explanation
Average enrolment	Average number of parents that enrol each time the programme is run
Average completion rate	Average percentage of enrolled parents that complete the programme, defined as attending at least 5 sessions
Average additional portions of fruit and veg eaten per day	The average additional portions of fruit and vegetables eaten each day by child(ren) whose parents complete the programme
Hours of staff time per week	Average amount of staff time each week needed to run the programme
Setup cost	Cost of setting up the programme, including materials, training, wages, etc.
Annual running cost	Annual cost of running the programme, including materials, training, wages, etc.

Here is an example question:

Question 1

Out of the two programmes below, which would you most prefer to see implemented?

	Programme A	*Programme B*
**Average enrollment**	**10 parents**	**6 parents**
**Average completion rate (attending at least 5 sessions)**	**50% (5 parents)**	**75% (4.5 parents)**
**Average additional portions of fruit & veg eaten per day**	**1 portion**	**2 portions**
**Hours of staff time per week**	**4 hours**	**12 hours**
**Setup cost**	**£20,000**	**£15,000**
**Annual running cost**	**£30,000**	**£30,000**

I would prefer:

○ Programme A ○ Programme B

Imagine one of these two programmes was going to be implemented. Simply indicate which you would prefer to be implemented.

There are 10 questions in this part of the survey.

Question 1

Out of the two programmes below, which would you most prefer to see implemented?

	Programme A	Programme B
Average enrollment	6 parents	8 parents
Average completion rate (attending at least 5 sessions)	80% (4.8 parents)	75% (6 parents)
Average additional portions of fruit & veg eaten per day	2 portions	0.5 portions
Hours of staff time per week	4 hours	12 hours
Setup cost	£15,000	£30,000
Annual running cost	£30,000	£15,000

I would prefer:

- ○ Programme A
- ○ Programme B

Question 2

Out of the two programmes below, which would you most prefer to see implemented?

	Programme A	Programme B
Average enrollment	10 parents	10 parents
Average completion rate (attending at least 5 sessions)	50% (5 parents)	80% (8 parents)
Average additional portions of fruit & veg eaten per day	0.5 portions	2 portions
Hours of staff time per week	4 hours	12 hours
Setup cost	£15,000	£30,000
Annual running cost	£20,000	£30,000

I would prefer:

○ Programme A ○ Programme B

Question 3

Out of the two programmes below, which would you most prefer to see implemented?

	Programme A	Programme B
Average enrollment	6 parents	10 parents
Average completion rate (attending at least 5 sessions)	80% (4.8 parents)	80% (8 parents)
Average additional portions of fruit & veg eaten per day	0.5 portions	2 portions
Hours of staff time per week	4 hours	12 hours
Setup cost	£30,000	£20,000
Annual running cost	£20,000	£30,000

I would prefer:

- ○ Programme A
- ○ Programme B

Question 4

Out of the two programmes below, which would you most prefer to see implemented?

	Programme A	Programme B
Average enrollment	6 parents	8 parents
Average completion rate (attending at least 5 sessions)	50% (3 parents)	80% (6.4 parents)
Average additional portions of fruit & veg eaten per day	1 portion	2 portions
Hours of staff time per week	4 hours	12 hours
Setup cost	£30,000	£15,000
Annual running cost	£30,000	£15,000

I would prefer:

- Programme A
- Programme B

Question 5

Out of the two programmes below, which would you most prefer to see implemented?

	Programme A	Programme B
Average enrollment	10 parents	6 parents
Average completion rate (attending at least 5 sessions)	50% (5 parents)	50% (3 parents)
Average additional portions of fruit & veg eaten per day	0.5 portions	1 portion
Hours of staff time per week	12 hours	4 hours
Setup cost	£15,000	£30,000
Annual running cost	£30,000	£20,000

I would prefer:

○ Programme A ○ Programme B

Question 6

Out of the two programmes below, which would you most prefer to see implemented?

	Programme A	Programme B
Average enrollment	6 parents	10 parents
Average completion rate (attending at least 5 sessions)	80% (4.8 parents)	80% (8 parents)
Average additional portions of fruit & veg eaten per day	2 portions	0.5 portions
Hours of staff time per week	12 hours	12 hours
Setup cost	£15,000	£20,000
Annual running cost	£15,000	£30,000

I would prefer:

○ Programme A ○ Programme B

Question 7

Out of the two programmes below, which would you most prefer to see implemented?

	Programme A	Programme B
Average enrollment	6 parents	10 parents
Average completion rate (attending at least 5 sessions)	50% (3 parents)	50% (5 parents)
Average additional portions of fruit & veg eaten per day	0.5 portions	2 portions
Hours of staff time per week	12 hours	4 hours
Setup cost	£20,000	£20,000
Annual running cost	£20,000	£30,000

I would prefer:

○ Programme A ○ Programme B

Question 8

Out of the two programmes below, which would you most prefer to see implemented?

	Programme A	Programme B
Average enrollment	10 parents	6 parents
Average completion rate (attending at least 5 sessions)	75% (7.5 parents)	80% (4.8 parents)
Average additional portions of fruit & veg eaten per day	2 portions	0.5 portions
Hours of staff time per week	4 hours	12 hours
Setup cost	£30,000	£15,000
Annual running cost	£30,000	£15,000

I would prefer:

- ○ Programme A
- ○ Programme B

Question 9

Out of the two programmes below, which would you most prefer to see implemented?

	Programme A	Programme B
Average enrollment	6 parents	10 parents
Average completion rate (attending at least 5 sessions)	50% (3 parents)	50% (5 parents)
Average additional portions of fruit & veg eaten per day	1 portion	0.5 portions
Hours of staff time per week	12 hours	4 hours
Setup cost	£20,000	£30,000
Annual running cost	£30,000	£15,000

I would prefer:

- ○ Programme A
- ○ Programme B

Question 10

Out of the two programmes below, which would you most prefer to see implemented?

	Programme A	Programme B
Average enrollment	10 parents	6 parents
Average completion rate (attending at least 5 sessions)	80% (8 parents)	50% (3 parents)
Average additional portions of fruit & veg eaten per day	2 portions	1 portion
Hours of staff time per week	12 hours	4 hours
Setup cost	£15,000	£30,000
Annual running cost	£20,000	£15,000

I would prefer:

- ○ Programme A
- ○ Programme B

Part 2

In the final part, we would like to ask about you and your work.

What is your gender?

- Male
- Female
- Other/prefer not to say

How would you describe your ethnicity?

- White - English/Welsh/Scottish/Northern Irish/British
- White – Irish
- White – Gypsy or Irish Traveller
- White – Any other White background
- Mixed/Multiple ethnic group - White and Black Caribbean
- Mixed/Multiple ethnic group - White and Black African
- Mixed/Multiple ethnic group - White and Asian
- Mixed/Multiple ethnic group - Any other Mixed/Multiple ethnic background
- Asian/Asian British - Indian
- Asian/Asian British - Pakistani
- Asian/Asian British - Bangladeshi
- Asian/Asian British - Chinese
- Asian/Asian British - Any other Asian background
- Black/ African/Caribbean/Black British - African
- Black/ African/Caribbean/Black British – Caribbean
- Black/ African/Caribbean/Black British - Any other Black/African/Caribbean background
- Arab

- ○ Other

If you selected Other, please specify:

How would you describe your role? Select all which apply.

- ☐ I commission services
- ☐ I make recommendations to others about what services to commission.
- ☐ I help implement services
- ☐ Other

If you selected Other, please specify:

How long have you been in your current role?

Years:

Months:

In what area(s) of the country do you work? Select all that apply.

- [ ] North West England
- [ ] North East England
- [ ] Yorkshire and Humber
- [ ] West Midlands
- [ ] East Midlands
- [ ] East of England
- [ ] South West England
- [ ] South East England
- [ ] London
- [ ] Northern Ireland
- [ ] Wales
- [ ] Scotland
- [ ] Non-UK
- [ ] Other

If you selected Other, please specify:

End of survey

Thank you for taking the time to complete this survey.

Key for selection options

15 - Years:

0
1
2
3
4
5
6
7
8
9
10+

16 - Months:

0
1
2
3
4
5
6
7
8
9
10
11
12
